# Crystal stucture of methyl 2-({[2-(meth­oxy­carbon­yl)phen­yl]carbamo­yl}amino)­benzoate

**DOI:** 10.1107/S2056989015006465

**Published:** 2015-04-09

**Authors:** Hasna Yassine, Mostafa Khouili, Lahcen El Ammari, Mohamed Saadi, El Mostafa Ketatni

**Affiliations:** a Laboratoire de Chimie Organique et Analytique, Université Sultan Moulay Slimane, Faculté des Sciences et Techniques, BP 523, 23000 Béni-Mellal, Morocco; b Laboratoire de Chimie du Solide Appliquée, Faculté des Sciences, Université Mohammed V, Avenue Ibn Battouta, BP. 1014, Rabat, Morocco; c Laboratoire de Spectrochimie Applique et Environnement, Université Sultan Moulay Slimane, Faculté des Sciences et Techniques, BP 523, 23000 Béni-Mellal, Morocco

**Keywords:** crystal structure, urea derivative, hydrogen bonding

## Abstract

In the title compound, C_17_H_16_N_2_O_5_, the dihedral angles between the central urea [N—C(=O)—N] fragment and its attached benzene rings are 20.20 (14) and 24.24 (13)°; the dihedral angle between the aromatic rings is 42.1 (1)°. The mol­ecular conformation is consolidated by two intra­molecular N—H⋯O hydrogen bonds, which both generate *S*(6) rings. In the crystal, inversion dimers linked by pairs of C—H⋯O inter­actions generate *R*_2_^2^(14) loops. The dimers are linked by further C—H⋯O inter­actions into (011) sheets.

## Related literature   

For the medical and biological activities of urea derivatives, see: Abad *et al.* (2004 ▸); Chen *et al.* (2005 ▸); Batra *et al.* (2006 ▸). For cytokinin activity, see: Wang *et al.* (2001 ▸); Ricci *et al.* (2005 ▸).
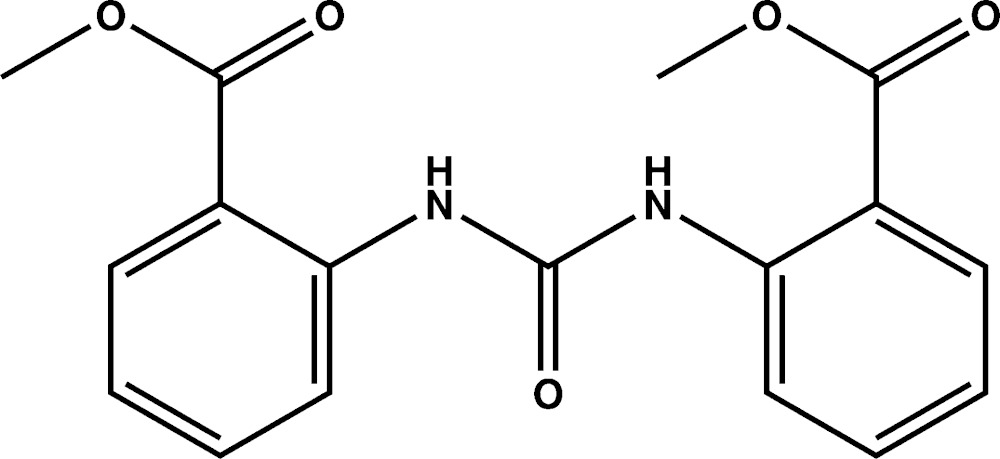


## Experimental   

### Crystal data  

C_17_H_16_N_2_O_5_
*M* *_r_* = 328.32
Monoclinic, 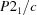

*a* = 9.005 (8) Å
*b* = 23.80 (2) Å
*c* = 7.400 (7) Å
β = 93.66 (4)°
*V* = 1583 (3) Å^3^
*Z* = 4
Mo *K*α radiation
μ = 0.10 mm^−1^
*T* = 296 K
0.42 × 0.36 × 0.29 mm

### Data collection  

Bruker X8 APEXII CCD diffractometer
Absorption correction: multi-scan (*SADABS*; Bruker, 2009 ▸) *T*_min_ = 0.693, *T*_max_ = 0.747
11500 measured reflections
3364 independent reflections
1667 reflections with *I* > 2σ(*I*)
*R*_int_ = 0.066

### Refinement  

*R*[*F* ^2^ > 2σ(*F* ^2^)] = 0.050
*wR*(*F* ^2^) = 0.137
*S* = 0.95
3364 reflections
220 parameters
H-atom parameters constrained
Δρ_max_ = 0.21 e Å^−3^
Δρ_min_ = −0.20 e Å^−3^

### 

Data collection: *APEX2* (Bruker, 2009 ▸); cell refinement: *SAINT* (Bruker, 2009 ▸); data reduction: *SAINT*; program(s) used to solve structure: *SHELXS97* (Sheldrick, 2008 ▸); program(s) used to refine structure: *SHELXL97* (Sheldrick, 2008 ▸); molecular graphics: *ORTEP-3 for Windows* (Farrugia, 2012 ▸); software used to prepare material for publication: *PLATON* (Spek, 2009 ▸) and *publCIF* (Westrip, 2010 ▸).

## Supplementary Material

Crystal structure: contains datablock(s) I. DOI: 10.1107/S2056989015006465/hb7393sup1.cif

Structure factors: contains datablock(s) I. DOI: 10.1107/S2056989015006465/hb7393Isup2.hkl

Supporting information file. DOI: 10.1107/S2056989015006465/hb7393Isup3.cml

**Fig. 1.**
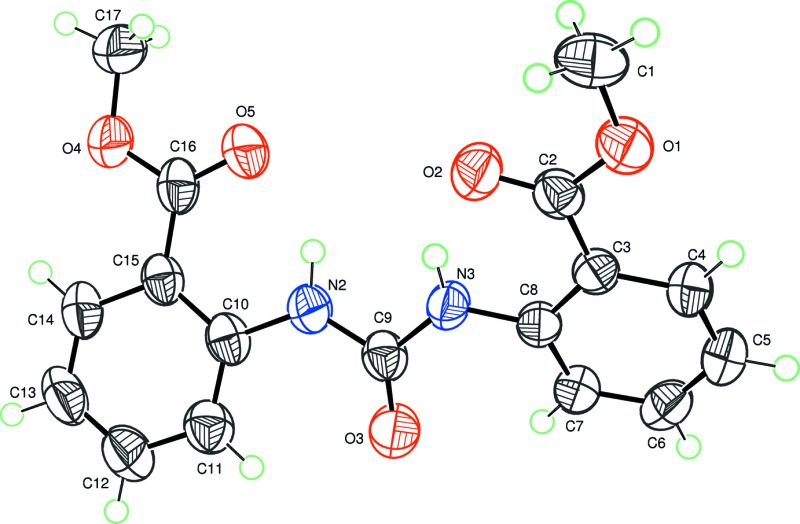
A view of the molecule of the title compound, showing displacement ellipsoids drawn at the 50% probability level.

. DOI: 10.1107/S2056989015006465/hb7393fig1.tif
A view of the mol­ecule of the title compound, showing displacement ellipsoids drawn at the 50% probability level.

**Fig. 2.**
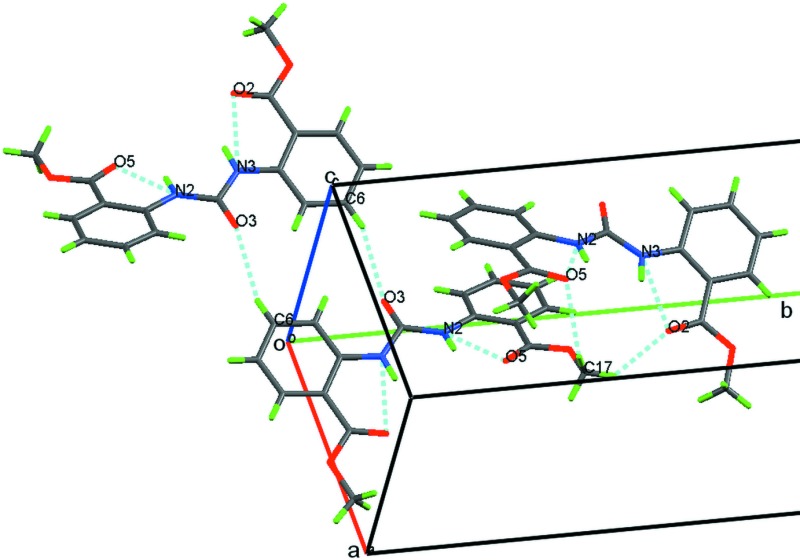
Part of the crystal structure of the title compound, showing hydrogen-bonded (dashed lines) dimers.

. DOI: 10.1107/S2056989015006465/hb7393fig2.tif
Part of the crystal structure of the title compound, showing hydrogen-bonded (dashed lines) dimers.

CCDC reference: 1056943

Additional supporting information:  crystallographic information; 3D view; checkCIF report

## Figures and Tables

**Table 1:** Hydrogen-bond geometry (, )

*D*H*A*	*D*H	H*A*	*D* *A*	*D*H*A*
N2H2*N*O5	0.86	1.96	2.677(3)	140
N3H3*N*O2	0.86	1.92	2.659(3)	144
C6H6O3^i^	0.93	2.57	3.442(4)	157
C17H17*C*O2^ii^	0.96	2.46	3.176(4)	132

Symmetry codes: (i) 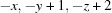; (ii) 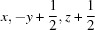.

## supplementary crystallographic information

### S1. Comment

Urea derivatives are very interesting reagents due to their useful properties and important medical and biological applications, such as insecticidal, fungicidal, herbicidal, anti-infectives and plant-growth regulating activities (Abad *et al.*, 2004; Chen *et al.*, 2005, Batra *et al.*, 2006), especially cytokinin activity (Wang *et al.*, 2001; Ricci *et al.*, 2005). Symmetrical disubstituted ureas generally form a polar hydrogen-bond chain, with anti NH donors and carbonyl O-atom acceptors in a bifurcated motif. As part of our studies in this area, the title compound C_17_H_16_N_2_O_5_ was synthesized and its crystal structure is reported in the present work.

The molecular structure of the title compound, a symmetrical urea derivative, is displayed in Fig. 1. This molecule is build up from two methyl benzoate linked through N–C(=O)–N fragment but not symmetric. The dihedral angle between the two phenyl rings (C3 to C8) and (C10 to C15) is of 42.1 (1)°.

In the crystal, the molecular conformation is stabilized by two intramolecular N—H···O hydrogen bonds and each molecule is linked to its symmetric by two intermolecular C—H···O hydrogen bonds to form centrosymmetric dimers as shown in Fig.2.

### S2. Experimental

A solution of 2-(methoxycarbonyl)benzoic acid (100 mg, 0.56 mmol), DPPA (0.194 ml, 0.90 mmol) and Et_3_N (0.127 ml, 0.90 mmol) in toluene (3 ml) was refluxed for 4 h. After cooling to room temperature, the reaction mixture was concentrated. The residue was purified by column chromatography using EtOAc-Hexane (1:9 *v*/*v*) as eluent to give yellow blocks with yield = 40% and m.p. = 411 K.

### S3. Refinement

All H atoms could be located in a difference Fourier map. However, they were placed in calculated positions with C–H = 0.93–0.96 Å; N—H = 0.86 Å, and refined as riding on their parent atoms with *U*_iso_(H) = 1.2 *U*_eq_ for aromatic, C—H, N–H and *U*_iso_(H) = 1.5 *U*_eq_ for methyl. Two outlier (1 0 0) and (0 2 0) was omitted in the last cycles of refinement.

### Crystal data

**Table:**

C_17_H_16_N_2_O_5_	*F*(000) = 688
*M**_r_* = 328.32	*D*_x_ = 1.378 Mg m^−^^3^
Monoclinic, *P*2_1_/*c*	Mo *K*α radiation, λ = 0.71073 Å
Hall symbol: -P 2ybc	Cell parameters from 3364 reflections
*a* = 9.005 (8) Å	θ = 2.4–26.7°
*b* = 23.80 (2) Å	µ = 0.10 mm^−^^1^
*c* = 7.400 (7) Å	*T* = 296 K
β = 93.66 (4)°	Block, yellow
*V* = 1583 (3) Å^3^	0.42 × 0.36 × 0.29 mm
*Z* = 4	

### Data collection

**Table:**

Bruker X8 APEXII CCD diffractometer	3364 independent reflections
Radiation source: fine-focus sealed tube	1667 reflections with *I* > 2σ(*I*)
Graphite monochromator	*R*_int_ = 0.066
φ and ω scans	θ_max_ = 26.7°, θ_min_ = 2.4°
Absorption correction: multi-scan (*SADABS*; Bruker, 2009)	*h* = −11→7
*T*_min_ = 0.693, *T*_max_ = 0.747	*k* = −29→30
11500 measured reflections	*l* = −9→9

### Refinement

**Table:**

Refinement on *F*^2^	Secondary atom site location: difference Fourier map
Least-squares matrix: full	Hydrogen site location: inferred from neighbouring sites
*R*[*F*^2^ > 2σ(*F*^2^)] = 0.050	H-atom parameters constrained
*wR*(*F*^2^) = 0.137	*w* = 1/[σ^2^(*F*_o_^2^) + (0.0578*P*)^2^] where *P* = (*F*_o_^2^ + 2*F*_c_^2^)/3
*S* = 0.95	(Δ/σ)_max_ < 0.001
3364 reflections	Δρ_max_ = 0.21 e Å^−^^3^
220 parameters	Δρ_min_ = −0.20 e Å^−^^3^
0 restraints	Extinction correction: *SHELXL97* (Sheldrick, 2008), Fc^*^=kFc[1+0.001xFc^2^λ^3^/sin(2θ)]^-1/4^
Primary atom site location: structure-invariant direct methods	Extinction coefficient: 0.0052 (11)

### Special details

**Table:**

Geometry. All e.s.d.'s (except the e.s.d. in the dihedral angle between two l.s. planes) are estimated using the full covariance matrix. The cell e.s.d.'s are taken into account individually in the estimation of e.s.d.'s in distances, angles and torsion angles; correlations between e.s.d.'s in cell parameters are only used when they are defined by crystal symmetry. An approximate (isotropic) treatment of cell e.s.d.'s is used for estimating e.s.d.'s involving l.s. planes.
Refinement. Refinement of *F*^2^ against all reflections. The weighted *R*-factor *wR* and goodness of fit *S* are based on *F*^2^, conventional *R*-factors *R* are based on *F*, with *F* set to zero for negative *F*^2^. The threshold expression of *F*^2^ > σ(*F*^2^) is used only for calculating *R*-factors(gt) *etc*. and is not relevant to the choice of reflections for refinement. *R*-factors based on *F*^2^ are statistically about twice as large as those based on *F*, and *R*- factors based on all data will be even larger.

### Fractional atomic coordinates and isotropic or equivalent isotropic displacement parameters (Å^2^)

**Table:**

	*x*	*y*	*z*	*U*_iso_*/*U*_eq_	
C1	0.8216 (3)	0.50547 (11)	0.6105 (4)	0.0617 (8)	
H1A	0.8796	0.5351	0.5624	0.093*	
H1B	0.8023	0.4772	0.5194	0.093*	
H1C	0.8755	0.4891	0.7136	0.093*	
C2	0.5813 (3)	0.49037 (10)	0.7155 (3)	0.0413 (6)	
C3	0.4424 (2)	0.51598 (9)	0.7748 (3)	0.0370 (6)	
C4	0.4261 (3)	0.57446 (9)	0.7763 (3)	0.0463 (6)	
H4	0.5028	0.5968	0.7377	0.056*	
C5	0.3000 (3)	0.59993 (10)	0.8334 (3)	0.0524 (7)	
H5	0.2913	0.6389	0.8324	0.063*	
C6	0.1861 (3)	0.56676 (10)	0.8925 (3)	0.0496 (7)	
H6	0.1012	0.5837	0.9328	0.060*	
C7	0.1975 (3)	0.50878 (9)	0.8921 (3)	0.0421 (6)	
H7	0.1199	0.4871	0.9315	0.050*	
C8	0.3249 (3)	0.48226 (9)	0.8331 (3)	0.0377 (6)	
C9	0.2326 (3)	0.38375 (10)	0.8583 (3)	0.0429 (6)	
C10	0.2281 (3)	0.27870 (9)	0.8808 (3)	0.0420 (6)	
C11	0.0793 (3)	0.27180 (10)	0.8152 (3)	0.0519 (7)	
H11	0.0219	0.3031	0.7827	0.062*	
C12	0.0181 (3)	0.21894 (11)	0.7988 (4)	0.0607 (8)	
H12	−0.0800	0.2150	0.7535	0.073*	
C13	0.0991 (3)	0.17166 (11)	0.8482 (4)	0.0627 (8)	
H13	0.0560	0.1362	0.8371	0.075*	
C14	0.2450 (3)	0.17759 (10)	0.9142 (3)	0.0511 (7)	
H14	0.2997	0.1457	0.9478	0.061*	
C15	0.3129 (3)	0.23038 (9)	0.9319 (3)	0.0407 (6)	
C16	0.4711 (3)	0.23461 (10)	1.0022 (3)	0.0460 (6)	
C17	0.6894 (3)	0.18555 (11)	1.1114 (4)	0.0695 (9)	
H17A	0.6980	0.2026	1.2292	0.104*	
H17B	0.7470	0.2066	1.0300	0.104*	
H17C	0.7258	0.1477	1.1196	0.104*	
N2	0.2964 (2)	0.33173 (7)	0.8915 (3)	0.0472 (5)	
H2N	0.3903	0.3306	0.9209	0.057*	
N3	0.3414 (2)	0.42374 (7)	0.8385 (3)	0.0443 (5)	
H3N	0.4311	0.4140	0.8169	0.053*	
O1	0.68143 (18)	0.52810 (7)	0.6650 (2)	0.0536 (5)	
O2	0.60738 (18)	0.44002 (7)	0.7108 (2)	0.0557 (5)	
O3	0.0994 (2)	0.39320 (7)	0.8476 (3)	0.0692 (6)	
O4	0.5330 (2)	0.18523 (7)	1.0436 (3)	0.0618 (5)	
O5	0.5419 (2)	0.27802 (7)	1.0207 (3)	0.0710 (6)	

### Atomic displacement parameters (Å^2^)

**Table:**

	*U*^11^	*U*^22^	*U*^33^	*U*^12^	*U*^13^	*U*^23^
C1	0.0386 (16)	0.080 (2)	0.0678 (19)	−0.0038 (14)	0.0124 (14)	0.0022 (15)
C2	0.0424 (15)	0.0422 (15)	0.0387 (14)	−0.0047 (12)	−0.0025 (12)	0.0035 (11)
C3	0.0377 (14)	0.0362 (13)	0.0364 (13)	−0.0004 (11)	−0.0018 (11)	0.0031 (10)
C4	0.0503 (16)	0.0346 (13)	0.0536 (16)	−0.0020 (12)	0.0010 (13)	0.0064 (11)
C5	0.0583 (18)	0.0350 (14)	0.0638 (18)	0.0053 (13)	0.0029 (14)	−0.0007 (12)
C6	0.0504 (16)	0.0481 (15)	0.0501 (16)	0.0133 (13)	0.0017 (13)	−0.0014 (12)
C7	0.0438 (15)	0.0390 (14)	0.0435 (15)	0.0014 (12)	0.0039 (12)	0.0005 (11)
C8	0.0414 (14)	0.0359 (13)	0.0352 (13)	0.0003 (11)	−0.0029 (11)	0.0003 (10)
C9	0.0443 (16)	0.0388 (14)	0.0465 (15)	−0.0055 (12)	0.0091 (12)	0.0008 (11)
C10	0.0484 (16)	0.0390 (14)	0.0396 (14)	−0.0089 (12)	0.0111 (12)	0.0001 (10)
C11	0.0528 (18)	0.0471 (16)	0.0561 (17)	−0.0068 (13)	0.0063 (14)	0.0036 (12)
C12	0.0591 (19)	0.0541 (18)	0.0686 (19)	−0.0156 (15)	0.0011 (15)	−0.0027 (14)
C13	0.066 (2)	0.0463 (16)	0.076 (2)	−0.0211 (15)	0.0036 (16)	−0.0101 (14)
C14	0.0626 (19)	0.0346 (14)	0.0570 (17)	−0.0066 (13)	0.0117 (14)	−0.0041 (12)
C15	0.0467 (15)	0.0362 (13)	0.0404 (14)	−0.0069 (11)	0.0115 (12)	−0.0024 (10)
C16	0.0584 (18)	0.0328 (14)	0.0480 (16)	−0.0052 (13)	0.0128 (13)	0.0008 (11)
C17	0.0476 (18)	0.0535 (17)	0.107 (3)	0.0005 (14)	0.0035 (17)	0.0059 (16)
N2	0.0441 (12)	0.0362 (11)	0.0614 (14)	−0.0054 (10)	0.0046 (11)	0.0055 (10)
N3	0.0384 (12)	0.0321 (11)	0.0632 (14)	0.0010 (9)	0.0094 (10)	0.0043 (9)
O1	0.0435 (11)	0.0497 (10)	0.0688 (12)	−0.0056 (8)	0.0134 (9)	0.0033 (8)
O2	0.0486 (11)	0.0416 (10)	0.0780 (13)	0.0030 (9)	0.0137 (9)	−0.0019 (9)
O3	0.0417 (11)	0.0492 (11)	0.1177 (18)	−0.0013 (9)	0.0138 (11)	−0.0042 (10)
O4	0.0544 (12)	0.0369 (10)	0.0938 (15)	−0.0016 (9)	0.0033 (10)	0.0022 (9)
O5	0.0606 (13)	0.0380 (11)	0.1121 (17)	−0.0105 (9)	−0.0126 (12)	0.0073 (10)

### Geometric parameters (Å, º)

**Table:**

C1—O1	1.453 (3)	C10—N2	1.404 (3)
C1—H1A	0.9600	C10—C11	1.405 (4)
C1—H1B	0.9600	C10—C15	1.418 (3)
C1—H1C	0.9600	C11—C12	1.376 (3)
C2—O2	1.222 (3)	C11—H11	0.9300
C2—O1	1.342 (3)	C12—C13	1.377 (4)
C2—C3	1.483 (3)	C12—H12	0.9300
C3—C4	1.400 (3)	C13—C14	1.379 (4)
C3—C8	1.417 (3)	C13—H13	0.9300
C4—C5	1.378 (3)	C14—C15	1.400 (3)
C4—H4	0.9300	C14—H14	0.9300
C5—C6	1.387 (3)	C15—C16	1.489 (4)
C5—H5	0.9300	C16—O5	1.217 (3)
C6—C7	1.384 (3)	C16—O4	1.328 (3)
C6—H6	0.9300	C17—O4	1.465 (3)
C7—C8	1.403 (3)	C17—H17A	0.9600
C7—H7	0.9300	C17—H17B	0.9600
C8—N3	1.401 (3)	C17—H17C	0.9600
C9—O3	1.218 (3)	N2—H2N	0.8600
C9—N2	1.381 (3)	N3—H3N	0.8646
C9—N3	1.381 (3)		
			
O1—C1—H1A	109.5	C12—C11—C10	120.3 (2)
O1—C1—H1B	109.5	C12—C11—H11	119.8
H1A—C1—H1B	109.5	C10—C11—H11	119.8
O1—C1—H1C	109.5	C11—C12—C13	121.4 (3)
H1A—C1—H1C	109.5	C11—C12—H12	119.3
H1B—C1—H1C	109.5	C13—C12—H12	119.3
O2—C2—O1	121.0 (2)	C12—C13—C14	119.1 (2)
O2—C2—C3	125.3 (2)	C12—C13—H13	120.5
O1—C2—C3	113.7 (2)	C14—C13—H13	120.5
C4—C3—C8	118.7 (2)	C13—C14—C15	121.7 (2)
C4—C3—C2	120.1 (2)	C13—C14—H14	119.2
C8—C3—C2	121.2 (2)	C15—C14—H14	119.2
C5—C4—C3	121.9 (2)	C14—C15—C10	118.6 (2)
C5—C4—H4	119.0	C14—C15—C16	119.7 (2)
C3—C4—H4	119.0	C10—C15—C16	121.7 (2)
C4—C5—C6	119.1 (2)	O5—C16—O4	121.1 (3)
C4—C5—H5	120.4	O5—C16—C15	125.4 (2)
C6—C5—H5	120.4	O4—C16—C15	113.5 (2)
C7—C6—C5	120.7 (2)	O4—C17—H17A	109.5
C7—C6—H6	119.6	O4—C17—H17B	109.5
C5—C6—H6	119.6	H17A—C17—H17B	109.5
C6—C7—C8	120.8 (2)	O4—C17—H17C	109.5
C6—C7—H7	119.6	H17A—C17—H17C	109.5
C8—C7—H7	119.6	H17B—C17—H17C	109.5
N3—C8—C7	121.7 (2)	C9—N2—C10	128.3 (2)
N3—C8—C3	119.4 (2)	C9—N2—H2N	117.6
C7—C8—C3	118.7 (2)	C10—N2—H2N	114.0
O3—C9—N2	125.1 (2)	C9—N3—C8	127.8 (2)
O3—C9—N3	124.7 (2)	C9—N3—H3N	120.8
N2—C9—N3	110.3 (2)	C8—N3—H3N	111.1
N2—C10—C11	122.0 (2)	C2—O1—C1	116.1 (2)
N2—C10—C15	119.2 (2)	C16—O4—C17	117.03 (19)
C11—C10—C15	118.8 (2)		
			
O2—C2—C3—C4	179.2 (2)	C13—C14—C15—C16	−179.4 (2)
O1—C2—C3—C4	−0.7 (3)	N2—C10—C15—C14	−177.6 (2)
O2—C2—C3—C8	0.1 (3)	C11—C10—C15—C14	0.3 (3)
O1—C2—C3—C8	−179.83 (19)	N2—C10—C15—C16	2.0 (3)
C8—C3—C4—C5	0.3 (3)	C11—C10—C15—C16	179.9 (2)
C2—C3—C4—C5	−178.9 (2)	C14—C15—C16—O5	178.9 (2)
C3—C4—C5—C6	0.5 (4)	C10—C15—C16—O5	−0.8 (4)
C4—C5—C6—C7	−0.9 (4)	C14—C15—C16—O4	−0.5 (3)
C5—C6—C7—C8	0.4 (3)	C10—C15—C16—O4	179.9 (2)
C6—C7—C8—N3	177.4 (2)	O3—C9—N2—C10	15.1 (4)
C6—C7—C8—C3	0.4 (3)	N3—C9—N2—C10	−164.8 (2)
C4—C3—C8—N3	−177.8 (2)	C11—C10—N2—C9	6.1 (4)
C2—C3—C8—N3	1.4 (3)	C15—C10—N2—C9	−176.1 (2)
C4—C3—C8—C7	−0.8 (3)	O3—C9—N3—C8	11.5 (4)
C2—C3—C8—C7	178.4 (2)	N2—C9—N3—C8	−168.6 (2)
N2—C10—C11—C12	177.0 (2)	C7—C8—N3—C9	16.1 (3)
C15—C10—C11—C12	−0.9 (4)	C3—C8—N3—C9	−167.0 (2)
C10—C11—C12—C13	1.0 (4)	O2—C2—O1—C1	−1.7 (3)
C11—C12—C13—C14	−0.5 (4)	C3—C2—O1—C1	178.3 (2)
C12—C13—C14—C15	−0.2 (4)	O5—C16—O4—C17	−0.2 (4)
C13—C14—C15—C10	0.2 (4)	C15—C16—O4—C17	179.1 (2)

### Hydrogen-bond geometry (Å, º)

**Table:**

*D*—H···*A*	*D*—H	H···*A*	*D*···*A*	*D*—H···*A*
N2—H2*N*···O5	0.86	1.96	2.677 (3)	140
N3—H3*N*···O2	0.86	1.92	2.659 (3)	144
C6—H6···O3^i^	0.93	2.57	3.442 (4)	157
C17—H17*C*···O2^ii^	0.96	2.46	3.176 (4)	132

Symmetry codes: (i) −*x*, −*y*+1, −*z*+2; (ii) *x*, −*y*+1/2, *z*+1/2.
