# Supplementary figures and images for: Crystal stucture of methyl 2-({[2-(meth­oxy­carbon­yl)phen­yl]carbamo­yl}amino)­benzoate
Source: Acta Crystallogr E Crystallogr Commun. 2015 Apr 9;71(Pt 5):o297–8. doi: 10.1107/S2056989015006465 (PMC4420073; doi:10.1107/S2056989015006465)

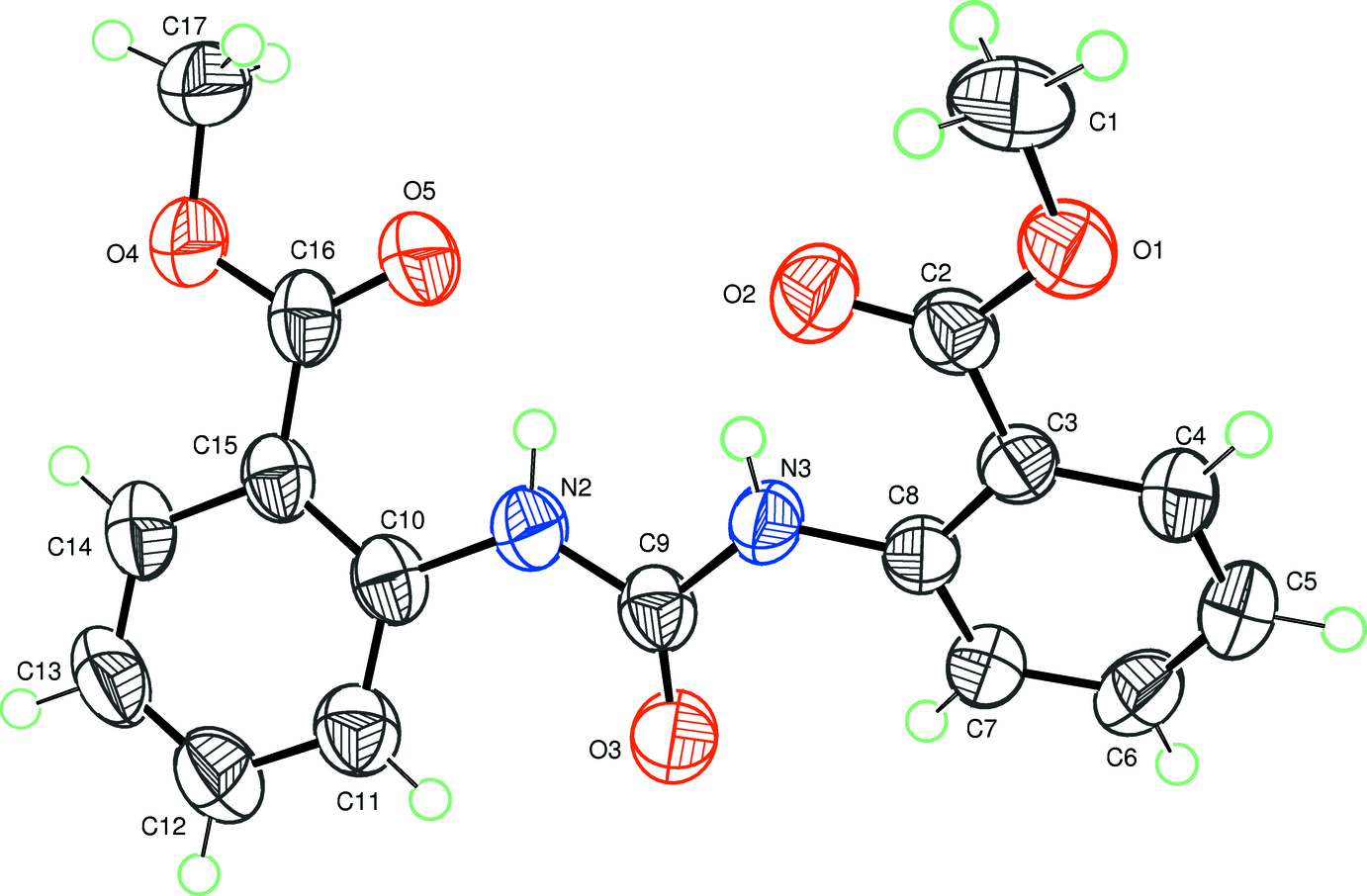

Supplement: Supplementary file 4 [file e-71-0o297-fig1.tif]

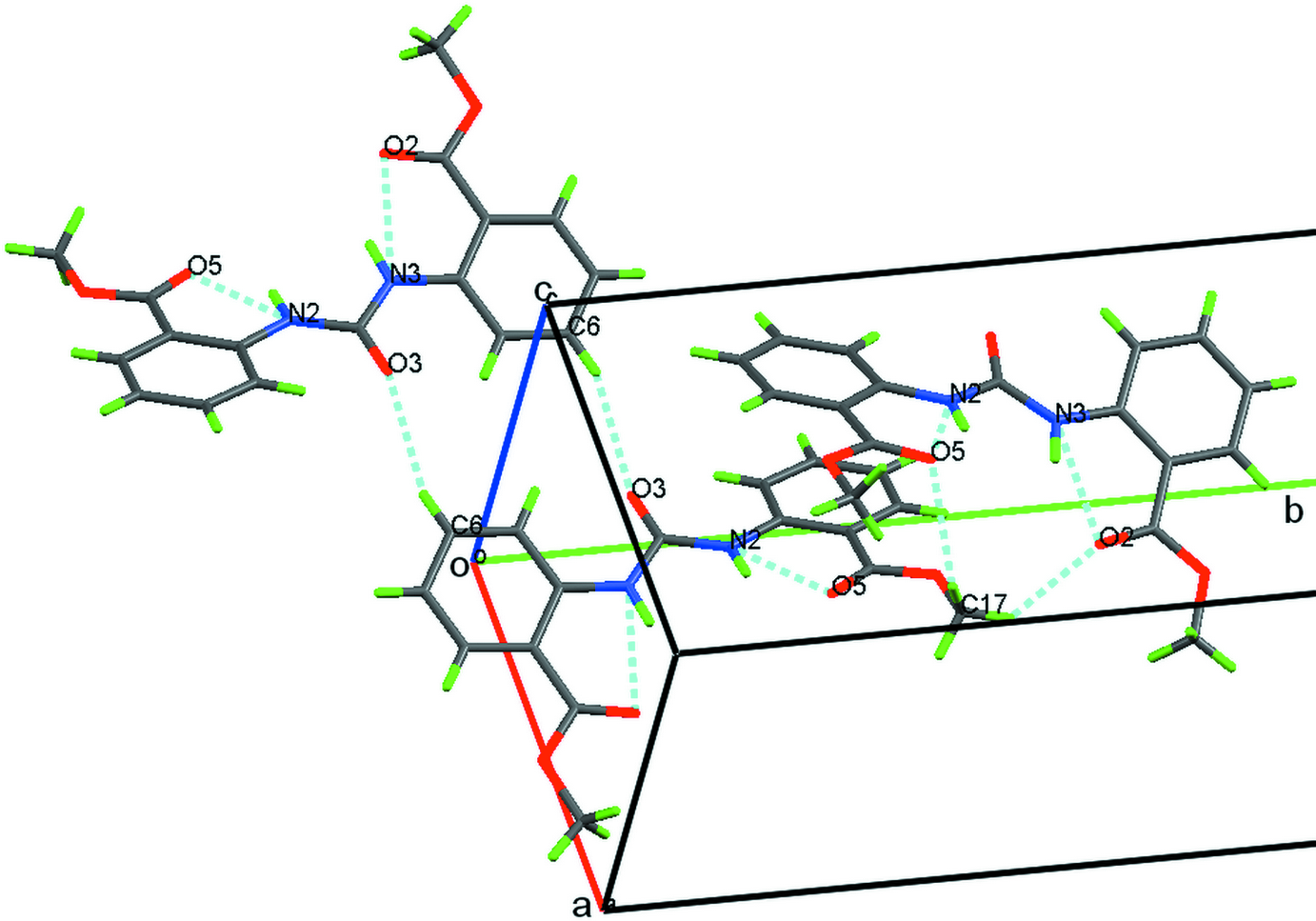

Supplement: Supplementary file 5 [file e-71-0o297-fig2.tif]
